# The Potential of Sun-Dried Grape Pomace as a Multi-Functional Ingredient for Herbal Infusion: Effects of Brewing Parameters on Composition and Bioactivity

**DOI:** 10.3390/antiox10040586

**Published:** 2021-04-10

**Authors:** Vlasios Goulas, Konstantina Stavrou, Christodoulos Michael, George Botsaris, Alexandra Barbouti

**Affiliations:** 1 Department of Agricultural Sciences, Biotechnology and Food Science, Cyprus University of Technology, 3603 Lemesos, Cyprus; konstantina.stavrou@outlook.com (K.S.); christodoulosmichael20@gmail.com (C.M.); george.botsaris@cut.ac.cy (G.B.); 2 Department of Anatomy-Histology-Embryology, Faculty of Medicine, University of Ioannina, 45110 Ioannina, Greece; abarbout@uoi.gr

**Keywords:** phenolics, antioxidant activity, antimicrobial activity, brewing, response surface methodology, digestive enzymes inhibitors

## Abstract

Wine and by-products are essential elements of a Mediterranean diet and considered as a reservoir of bioactive compounds with various health effects. Grape pomace, an easily available natural material of low cost, shares a similar wealth of health benefiting bioactive phytochemicals. The objective of this study was to explore the utilization of grape pomace from Commandaria dessert wine as main ingredient for functional infusions. Therefore, the ratio of water to grape pomace powder (40–200 mL g^−1^), infusion time (3–15 min) and temperature (55–95 °C) were optimized in terms of composition and bioactivity. Multiple response optimization indicated that brewing 200 mL water per g of material for 12.2 min at 95 °C, was the optimum method for preparing the infusion. Results also revealed a significant impact of three parameters as well as quadratic and interactive effects on composition and bioactivity of infusions. Furthermore, the infusion presents antimicrobial effects against *Listeria monocytogenes* serotypes and other common food pathogenic bacteria. Finally, a sensory evaluation was performed to assess the organoleptic attributes of the infusion and its improvement, with the addition of Mediterranean aromatic plants. Overall, the present work describes a promising strategy for the re-use of sun-dried grape pomace as a functional ingredient of infusions.

## 1. Introduction

The Mediterranean diet has generally been associated with a decreased risk of developing different chronic diseases and longer life expectancy. Antioxidants and fiber are considered as two main pillars of the Mediterranean diet and are key functional nutrients for healthy eating [1]. These findings motivated researchers to study Mediterranean fruits, vegetables and their products in order to discover bioactive phytochemicals with a potential to evolve into preventive and possibly therapeutic agents [2]. Moreover, their post-harvest processing and production of derivatives generates significant amounts of wastes that contain similar functional ingredients. Thus, the utilization of unexploited agricultural wastes to recover bioactive compounds is strongly recommended. This strategy fulfils the need to move towards healthy foods and foster environmental sustainability [3,4]. Among agricultural waste, the grape pomace is a rich source of bioactive compounds and many applications in food, cosmetic and pharmaceutical industry have been demonstrated [5].

The grape pomace comprises a variety of bioactive compounds at high content; especially phenolic acids, flavonols, flavanols, anthocyanins, tannins and stilbenes. The phenolic fraction of grape pomace is influenced by genetic factors, pre- and post-harvest treatments and winemaking techniques [6]. Its functional constituents are linked with many biological activities such antimicrobial, anti-viral, antioxidant, anti-inflammatory and anti-ageing effects and are also effective for the treatment of chronic diseases such as cancer, cardiovascular diseases, and metabolic syndrome [5,7,8]. Therefore, the utilization of grape pomace as functional constituent to produce innovative foods or to enrich existing foods has been studied thoroughly. More specifically, it has been used to prevent lipid oxidation and formation of hazard products e.g., acrylamide, to inhibit the food spoilage, to improve color and texture in many cases such as bakery, meat, and dairy products [5]. It has also been added as functional ingredient in dairy products [9,10], cereal bars, bakery products [11], pastas [12] and herbal beverages [13]. Furthermore, previous studies demonstrated the preparation of grape pomace beverages with anti-influenza and anti-inflammatory activity [14].

In Cyprus, the wine industry is one of the most important agri-food sectors yielding large amounts of wastes in a short period. The valorization of grape pomace to produce high-added value products is an emerging challenge to reduce environmental footprint and promote sustainability of wine production. “Xinisteri” white grape variety is widely cultivated and covers about 30% of Cypriot vineyards. It produces white wines and dessert wine ‘Commandaria’, a protected designation of origin product. The “Xinisteri” grapes are sun-dried to produce ‘Commandaria’, thus, this pomace contains high contents of bioactive polyphenols due to preconcentration [15]. Therefore, the objective of this study was to develop a functional infusion from ‘Commandaria’ grape pomace promoting potential physiological benefits. In this attempt, the brewing parameters as ratio of water to grape pomace powder, infusion time and temperature were optimized to prepare infusions with high content of bioactive compounds and potent biological activity. Finally, the sensory evaluation of infusions formulated from grape pomace alone or with added Mediterranean aromatic plants were performed to manipulate the organoleptic characteristics of the infusion.

## 2. Materials and Methods

### 2.1. Sample and Reagents

This study was conducted on sun-dried grape pomace from “Xinisteri” cultivar (*Vitis vinifera* L.). It was provided from Tsiakkas Winery Ltd. winery (Pelendri, Cyprus), obtained as wine by-product after alcoholic fermentation and pressing; from sun-dried grapes for the production of dessert wine Commandaria on October 2018. Grape pomace was dried at 105 °C and pulverized using an electric grinder (Bestron AKM1405150W, Bestron Nederland BV, s-Hertogenbosch, The Netherlands). Furthermore, dried lemon balm (*Melissa officinalis*) and lemon verbena (*Aloysia citrodora*) were purchased from local market.

Standards of gallic acid (≥98.0%), caffeic acid (≥98.0%), rutin (≥94.0%), quercetin (≥95.0%), (+)-catechin hydrate (≥95.0%), and 6-hydroxy-2,5,7,8-tetramethylchroman-2-carboxylic acid (Trolox) (≥97.0%), were purchased from Sigma Aldrich (St. Louis, MO, USA). Methanol and ethanol were obtained by Carlo Erba Reagents (Milan, Italy) and other reagents were provided by Merck (Darmstadt, Germany).

### 2.2. Experimental Design and Preparation of Infusions

For the preparation of infusion, 2 g of powdered grape pomace were placed into teabags with string heal seal (non-woven fabrics, 5.5 × 7 cm) and macerated with deionized water in conical flasks. Different water to solid ratios (40–200 mL g^−1^), times (3–15 min) and temperatures (55–95 °C) were tested. After brewing, a volume of 50 mL of samples was centrifuged and stored at −20 °C until further analysis.

In this work, the central composite design (CCD) was employed for the optimization of brewing parameters for grape pomace infusions. The design involved 20 randomly assigned runs. Three variables were chosen as the responses in the designed experiment, and each of them was analyzed at five various levels. Both coded and natural independent variables are given in Table 1. The model proposed for each response of Y was:

Y = *β*_0_ + *β*_1_X_1_ + *β*_2_X_2_ + *β*_3_X_3_ + *β*_11_X_1_^2^ + *β*_22_X_2_^2^ + *β*_33_X_3_^2^ + *β*_12_X_1_X_2_ + *β*_13_X_1_X_3_ + *β*_23_X_2_X_3_ (1)

where Y is response, X_1_—infusion time, X_2_—water to solid ratio, X_3_—infusion temperature, *β*_0_—interception coefficient, *β*_1_, *β*_2_, and *β*_3_—linear terms, *β*_11_, *β*_22_ and *β*_33_—quadratic terms, *β*_12_, *β*_13_ and *β*_23_—interaction regression coefficient terms, respectively.

The software Minitab^®^ version 17 (Minitab Inc., State College, PA, USA) was used for experiment design, data analysis and determination of optimal conditions.

### 2.3. Spectrophotometric Determination and Classification of Phenolic Fraction

For the determination of total phenolics, an aliquot of diluted infusion (50 μL) was mixed with 50 μL of Folin Ciocalteu reagent (1:5, *v*/*v*) and 100 μL of sodium hydroxide solution (0.35 mol L^−1^) in each well. After 3 min, the absorbance of samples was read at 760 nm. A standard curve of gallic acid was prepared and results were expressed as mg gallic acid equivalents (GAE) L^−1^ infusion [16].

The determination of hydroxycinnamic acid derivatives was performed according to previous protocol with slight modifications [17]. An amount of infusion (50 μL) was mixed with 25 μL 0.1% (*v*/*v*) HCl–ethanol solution and 200 μL 2% (*v*/*v*) HCl–ethanol solution. Then, the absorbance was monitored at 320 nm and results were expressed as mg caffeic acid equivalents (CAE) L^−1^ infusion.

The determination of total flavonoids was performed by the mixing of 100 μL distilled water, 10 μL of 50 g L^−1^ sodium nitrite and 25 μL of infusions. After a period of 5 min, 15 μL of 100 g L^−1^ aluminum chloride was added to the mixture. Subsequently, 50 μL of 1 mol L^−1^ sodium hydroxide and 50 μL of deionized water were added after 6 min. The mixture was shaken for 30 s in the plate reader prior to absorbance measurement at 510 nm. Results were expressed as mg catechin equivalents (CE) L^−1^ infusion [16].

Tannin content of infusions was determined using the tannins precipitation with the methyl-cellulose method [18]. An aliquot of 150 μL of infusions was mixed with 800 μL of deionized water, 250 μL of 0.04% (*w*/*v*) methyl-cellulose and 750 μL of saturated ammonium sulphate. For the blank, the sample aliquot was replaced by water and the methyl-cellulose and the ammonium sulphate were not included. After 20 min of reaction, the samples were centrifuged and the absorbance was determined at 280 nm. Results were expressed as mg tannins L^−1^ infusion.

### 2.4. Assessment of 1,1-Diphenyl-2-picrylhydrazyl (DPPH) Radical Scavenging Activity

The radical scavenging activity of grape skin infusions was measured using microplate DPPH assay. More specific, 20 μL of infusions were mixed with 50 μL of methanol and 150 μL DPPH methanolic solution (0.2 mM). Then, the mixtures were incubated for 30 min; and the absorbance was read at 515 nm. Results were expressed as μmol Trolox equivalents L^−1^ infusion [16].

### 2.5. Assessment of Antioxidant Activity Using Lecithin Liposome System

Antioxidative activity of infusions in lecithin liposome model system was determined according to previous work with slight modifications [19]. At first, lecithin liposomes were prepared by suspending lecithin in deionized water at a concentration of 8 mg mL^−1^ by stirring, followed by sonification for 30 min in a sonication bath. An aliquot of diluted infusions (150 μL) was added to 200 μL of lecithin liposome system and the mixture was then sonicated for 2 min. To initiate the reaction, 100 μL of 0.15 mol L^−1^ cupric acetate was added and the mixture was shaken in the dark using an orbital shaker incubator. The oxidation in lecithin liposome system was monitored at 48 h by determining the formation of conjugated dienes. Thus, 0.2 g of reaction mixture was dissolved in 5 mL of methanol and conjugated dienes were measured as the increase in absorbance at 234 nm. Results were expressed μmol rutin equivalents (RE) L^−1^ infusion.

### 2.6. Determination of Protein Glycation Inhibiting Activity

The anti-glycation activity of infusion was determined using an in vitro protocol [20]. In brief, 2 mL of phosphate buffer solution (1.5 mol L^−1^, pH 7.4) containing 50 mg mL^−1^ and 0.8 mol L^−1^ D-glucose were mixed with 500 μL and incubated at 37 °C for 7 days. The mixture also contained 0.2 g L^−1^ NaN_3_ to assure an aseptic condition. After incubation, the fluorescent intensity (excitation, 330 nm; emission, 410 nm) was measured. Results were expressed in μmol RE L^−1^ infusion.

### 2.7. Determination of Inhibitory Effects of Infusions on α-Amylase and α-Glucosidase–Enzymes Related to Hyperglycemia

An aliquot of infusion of 500 μL was mixed with 250 μL of *α*-amylase (0.5 mg mL^−^^1^ in 0.02 mol L^−1^ sodium phosphate buffer, pH 6.9) and the mixture was pre-incubated at 25 °C for 10 min. Then, 250 μL of starch solution (1% *w*/*v*) in 0.02  mol L^−1^ sodium phosphate buffer (pH 6.9) was added and then further incubated at 25 °C for 10 min. Afterwards, 500 μL of dinitrosalicylic acid was added into the mixture; the mixture was then incubated at 95 °C for 5 min. After cooling, 30 μL of reaction mixture was diluted with 270 μL deionized water and the absorbance was read at 540 nm [21]. Results were expressed μmol RE L^−1^ infusion.

Infusions (100 μL) were incubated at 37 °C with 50 μL *α*-glucosidase from *Saccharomyces cerevisiae* (1.0 U mL^−^^1^ in 0.1 mol L^−1^ phosphate buffer, pH 6.8). After 10 min, 25 μL of p-nitrophenyl-*α*-*d*-glucopyranoside (PNG) 5 mmol L^−1^ was added. The mixture was allowed for 5 min and the absorbance was measured at 405 nm against a blank solution where PNG was replaced with 50 μL of buffer [22]. Results were expressed μmol RE L^−1^ infusion.

### 2.8. Determination of Antibacterial Activity

The inhibitory effect of different concentrations (125–2000 μg·mL^−1^) of lyophilized infusion and pure compounds (gallic acid, catechin and quercetin) on the growth of *L. monocytogenes* EGD (serotype 1/2a)*, L. monocytogenes* Scott A (serotype 4b), *L. monocytogenes* NCTC 4885 (serotype 4b), *L. monocytogenes* NCTC 4994 (serotype 4b), *L. monocytogenes* ATCC 23074 (serotype 4b), *L. monocytogenes* NCTC 1792 (serotype 4b), *L. monocytogenes* NCTC 7973 (serotype 1/2a)*, Staphylococcus aureus* ATCC 6538*, Salmonella enterica* subsp*. enterica serovarm*, *Enteritidis* NCTC 5188 and *Escherichia coli* ATCC 11775 was determined by the broth microdilution method [23]. An aliquot of overnight bacterial cultures (10 μL) was enriched with 90 μL of fresh broth and subsequently mixed with 100 μL of tested infusion or pure compounds. Microbial growth kinetic was recorded on a Multiskan^™^ GO Microplate Photometer (Thermo Fisher Scientific, Vantaa, Finland). Optical density was read at 600 nm by taking measurements every 30 min for 18 h in controlled conditions of 37 °C. An agitation for 10 s was performed to achieve homogeneous suspensions before each measurement. All experiments were performed in triplicate. The minimum inhibitory concentration (MIC) was defined as the lowest concentration of infusion or pure compound at which bacterial growth was inhibited compared to the control.

### 2.9. Sensory Evaluation of Infusions

The sensory evaluation of the herbal infusions was performed by 20 panelists (12 male; 8 female), aged between 18 and 24 years old. A previous study with some modification was used to evaluate the sensory properties of herbal infusions [24]. A volume of about 30 mL of each warm infusion (65 ± 5 °C), was served randomly to the panelists in a 50 mL transparent cup. The members of the sensory panel were required to rinse their mouths thoroughly with warm water (~65 °C) after each evaluation and wait no less than 90 s before the next tasting, to minimize possible carry-over effects. In an attempt to appreciate the full sensory character of the infusion the panel members were asked to hold about 10 mL sample in the mouth for 5 s and swallow it gradually and where necessary, the tasting was repeated. The infusions were evaluated for a total of six attributes; color, aroma, flavor, aftertaste, astringency and overall acceptability. The results were recorded using a five-point hedonic scale extending from 1 (dislike very much) to 5 (live very much). All the samples were coded and none of the panel members received any other information regarding the infusions tested.

### 2.10. Statistical Analysis

Statistical analysis was carried out using the software package SPSS v22.0 (SPSS Inc., Chicago, IL, USA) and the comparison of averages of each treatment was based on the analysis of variance (One-Way ANOVA) according to Duncan’s multiple range test at significance level 5%.

## 3. Results and Discussion

### 3.1. Effects of Brewing Parameters on Bioactive Composition of Infusions

The present work investigates the potential of sun-dried grape pomace to be used as ingredient to produce herbal infusions with antioxidant and anti-hyperglycemic effects. Previous studies correlated these effects with the presence of different classes of polyphenols [25]. Thus, the optimization of brewing parameters aims to maximize the phenolic compounds in grape pomace infusion. More specific, the phenolic (TPC), hydroxycinnamic (THC), flavonoids (TFC) and tannin (TTC) contents were determined (Table 2). TPC values of the infusions ranged between 554 and 2901 mg GAE L^−1^ infusion highlighting the great importance of brewing procedure. The highest TPC value was found for the infusion prepared using 160 mL water per gram powder for 12 min at 85 °C. On the other hand, the use 40 mL water per gram powder for 9 min at 75 °C yielded the lowest TPC value. Furthermore, results showed that all brewing parameters had a significant impact on TPC (Table 3, *p* > 0.05). Quadratic effect of temperature and a negative interactive effect between water/solid ratio and temperature were also substantial for phenolic contents in infusion.

A great variety in THC of infusions was also found (Table 2); especially TFC ranged from 35.0 to 116.4 mg CAE L^−1^ infusion. Similarly to TFC, the highest THC was achieved using 160 mL water per gram powder for 12 min at 85 °C. According to the CCD, only temperature had a linear effect on THC, whereas quadratic effects of time and water/solid ratio was found (Table 3, *p* > 0.05). The proposed model was found as significant and non-significant lack of fit demonstrating the suitability of model to describe the effects of brewing parameters on THC.

Results also showed that grape pomace infusions comprised important amounts of flavonoids that were strongly affected by brewing parameters. More specific, all parameters studied had a significant effect on TFC resulting a range between 42.9 and 84.2 mg CE L^−1^ infusion. The richest infusion in flavonoids was prepared by the infusion of 160 mL water per gram powder for 12 min at 85 °C (Table 2). CCD reveals the quadratic effect of water/solid ratio and interactive effects for water/solid ratio with extraction time and temperatures. The polynomial model was found as significant and non-significant lack of fit.

Finally, TTC of infusions were determined as grape tannins are correlated with bioactivity and astringency [26]. Results showed a linear and quadratic effects of temperature and water/solid ratio on tannin contents in infusions (Table 3). The fluctuation of TTCs in infusions was from 0 to 24.09 mg tannins L^−1^ infusion. The maximum tannin content in infusion was used for the optimization of brewing parameters due to their bioactivity, although their astringency could decrease the acceptance of infusion by consumers.

### 3.2. Effects of Brewing Parameters on Bioactivity of Infusions

The brewing parameters of sun-dried grape pomace infusion were optimized in order to boost its bioactivity. In particular, the impact of brewing time, temperature and water/solid ratio on physiological functions related to the antioxidant and antidiabetic effects was studied. Except for DPPH assay, results of other biological assays were expressed as rutin equivalents since its antioxidant and antidiabetic potency is well-established [27]. Firstly, the DPPH activity of infusions was determined revealing a wide range of value; namely, it ranged between 1078 and 4672 μmol Trolox L^−1^ infusion. The brewing of 200 mL of water per gram of material for 9 min at 75 °C produced infusion with the highest DPPH activity, followed by the infusion that was prepared by the use of 160 mL water per gram powder for 12 min at 85 °C. The latter infusion had the highest phenolic content among infusions studied. The linear effects of three processing variables were significant (*p* > 0.05) on DPPH values of the infusions as it was observed for TPC and TFC. Table 3 shows also that the model and lack of fit were significant (*p* > 0.05).

The antioxidant effect of infusions was also assessed using a lecithin liposome model. Similarly to DPPH assay, the most active sample was prepared by brewing of 200 mL of water per gram of material for 9 min at 75 °C. Surprisingly, data of this method showed a tendency slightly differing from that observed for DPPH values. This may be explained by different mechanisms of action of antioxidants. All measurements of the antioxidant effect of infusions on lecithin liposome model are displayed in Table 2. As it is seen, the antioxidant activity was in the range of 22.0–95.5 μmol RE L^−1^ infusion. According to Table 3, the antioxidant activity is strongly affected by all brewing parameters (*p* > 0.05). Furthermore, quadratic effect of water/solid ratio and interactive effects were also contributed to the polynomial model, which provides an acceptable prediction of three variables on antioxidant potency.

The anti-glycoxidative properties of raisin grape pomace infusions were assessed using a bovine serum albumin—glucose model. All brewing parameters had a significant effect on anti-glycoxidative activity of infusion (*p* > 0.05); this activity ranged between 0.262 and 2.336 μmol RE L^−1^ infusion (Table 2 and Table 3). The most active infusion was prepared by mixing 160 mL water per gram powder for 12 min at 85 °C, whereas the brewing of the same water to plant material ratio for 6 min at 65 °C yielded the infusion with the lowest BSA activity. Apart from brewing parameters, a quadratic effect of temperature and interactive effect of water/solid ratio and temperature were also found contributing to the polynomial model that was found as significant and non-significant lack of fit.

Finally, the inhibition effects of infusions on the α-glucosidase and the α-amylase activities were determined. Both assays are considered as a good indicator of antidiabetic activity and are used to screen phytoconstituents for potential drugs to treat diabetes mellitus [28]. A great diversity in inhibitory activities toward α-glucosidase and the α -amylase enzymes was observed (Table 2). More specifically, the inhibitory activity was 571 to 1720 μmol RE L^−1^ infusion and 80–389 μmol RE L^−1^ infusion for α-glucosidase and the α-amylase, respectively. The above diversity can be attributed to the fact that the inhibitory values were strongly affected by brewing time, temperature and water/solid ratio. According to Table 3, α-glucosidase inhibitory activity is also influenced by quadratic effects of water/solid ratio and temperature; while the corresponding activity of α-amylase was affected by quadratic effect of water/solid ratio and interactive effect between water/solid ratio and temperature. The brewing conditions for the preparation of the most adiabetic infusion were similar for both inhibitory effects.

### 3.3. Optimization of Parameters

The optimal conditions for the preparation of grape pomace infusion were determined after applying the desirability function for all of the investigated responses in terms of phenolic composition and bioactivity. The purpose of the optimization was to recover the maximum value of each response. Results showed that the brewing of 200 mL of water per gram of pomace powder for 9.8 min at 95 °C is the most appropriate method to prepare grape pomace infusion. Taking into consideration that the main objective of the study was to formulate a functional infusion, the optimal conditions were re-determined using the desirability function for responses related only to the bioactivity. In this case, the ratio water to solid and infusion temperature were same, but the optimum infusion time was 12.2 min. Afterwards, optimum conditions for preparation infusion with highest antioxidant or anti-hyperglycemic effects were also investigated, but there are no differences. Table 4 also demonstrates that the experimental values for optimized grape pomace infusion were close to predicted ones. This confirms that the proposed model was successfully applied for the brewing of grape pomace powder to obtain infusion with highest functionality.

### 3.4. Antimicrobial Effects of Infusion

The most active infusion was also tested for its antimicrobial effects against foodborne pathogenic bacteria. Previous studies present a significant bacterial growth inhibition by grape pomace extracts [29,30]. Therefore, the grape skin infusion was freeze-dried and its inhibitory effects against an array of *Listeria* serotypes, *Salmonella enterica*, *Staphylococcus aureus*, and *E. coli* was assessed. Results showed that minimum inhibitory concentration for the infusion against *Listeria* serotypes ranged between 0.25 and 2 mg mL^−1^ pointing out a variation in the susceptibility of different *L. monocytogenes* serotypes to infusion treatments. The anti-*Listeria* potential of the infusion is significantly higher than the potential of acetonic grape pomace extracts from four Virginia-grown grape varieties [30]. Although there is considerable fluctuation in the response of different *Listeria* strains to plant extracts, the increase of phenolic content in raisin grape pomace due to dehydration is possibly accountable for the improved growth inhibition of the present infusion compared to grape pomace extracts. The infusion also had an inhibitory effect on *S. enterica*, *S. aureus*, and *E. coli* bacteria (Table 5). In an attempt to rationalize the antimicrobial potency of infusion, the inhibitory effects of gallic acid, catechin and quercetin were determined. These compounds and their derivatives are substantial constituents of hydroxybenzoic acids, flavonols and flavan-3-ols in “Xinisteri” dehydrated grapes [15]. MIC values indicate that quercetin present the most potent antimicrobial activity among compounds studied (Table 5). Results also demonstrated that more phytochemicals contribute to the antimicrobial potency of infusion and synergistic interaction may occurs. Overall, the present work highlights the potential of the tested infusion to prevent the growth of some *Listeria* serotypes (MIC = 0.25–0.5 mg mL^−1^); at concentrations which are almost equal with a cup of infusion.

### 3.5. Sensory Analysis

**Figure 1:**
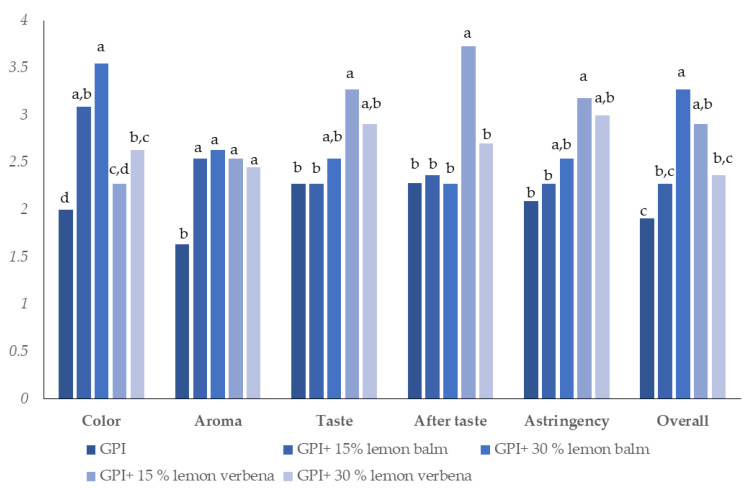
Mean values for sensory attributes of grape pomace infusions (GPI). Means with different letter are statistically different for each attribute.

The flavor and taste of grape pomace infusion was neutral and flat. These characteristics allow its consumption, but the consumers may not enjoy it. Thus, the improvement of organoleptic attributes of infusion were attempted using Mediterranean aromatic plants. More specifically, lemon balm and lemon verbena were added to grape pomace powder at a concentration of 15% *w*/*w* and 30% *w*/*w* due to its special flavor and taste as well as they are widely consumed as herbal infusions. Furthermore, both plant materials displayed antioxidant, antimicrobial and antidiabetic activity, thus, their addition was not expected to deplete significantly the functionality of infusion [31,32,33]. Figure 1 summarizes the scores for sensory evaluation attributes of five grape pomace infusions. It reveals a substantial impact of herbs on the infusion’s color. The addition of lemon balm into grape pomace powder contributed to the most attractive color among the tested infusions. Specifically, the slight yellow to straw color of pure grape pomace infusion was altered to a brown color. On the other hand, the enrichment of grape pomace infusion with lemon verbena yielded a dark green colored infusion. Regarding the aroma, all mixtures of herbs with grape pomace had a better acceptance than pure grape pomace infusions. About of 50% of participants found a sweet taste for all infusions, because of the use of sun-dried grape pomace and only 27% of participants stated the presence of grape’s aroma in pure grape pomace infusion (data not shown). Infusions with lemon balm smells like herbs; whereas the participants found aroma of herbs and fruits in infusions with lemon verbena. Similar findings were reported for taste of infusions; albeit the panelists preferred the taste of grape pomace-lemon verbana infusions. The addition of both herbs did not change statistically the astringency and aftertaste. Furthermore, the scores of overall acceptance of infusions shows that the mixtures of 30% lemon balm to grape pomace and 15% lemon verbena to grape pomace were preferred, for the preparation of infusions. Finally, all participants declared that the pure grape pomace infusion was pleasant but the supplementation with common herbs improved its organoleptic attributes. The latter is maybe correlated to the fact that the panelists are familiar with the consumption of these herbal infusions.

## 4. Conclusions

The present work highlights the potential of sun-dried grape pomace to be utilized as an ingredient for functional foods as herbal infusions. The market of these products is rapidly increasing and the food industry is constantly seeking ways to introduce new products and/or to reformulate traditional products. Therefore, the exploitation of sun-dried grape pomace to produce functional infusions is an alternative strategy to promote sustainability of wine-making and to create a novel product. This study describes the optimum brewing parameters to produce infusion with antioxidant, antimicrobial and anti-hyperglycemic effects and provides a guideline for its formulation. Finally, the neutral taste and aroma alongside the application of water as extractor, permit the use of the infusion or grape pomace infusion powder as an additive in many foods or edible coatings.

## Figures and Tables

**Table 1:** Central Composite Design with actual and values for brewing factors. The coded values of variables are given in parentheses.

Runs	Factors		
	Time (min)	Water/Solid Ratio (mL g^−1^)	Temperature (°C)
1	9 (0)	40 (−2)	75 (0)
2	9 (0)	120 (0)	55 (−2)
3	3 (−2)	120 (0)	75 (0)
4	9 (0)	200 (2)	75 (0)
5	15 (2)	120 (0)	75 (0)
6	9 (0)	120 (0)	75 (0)
7	9 (0)	120 (0)	75 (0)
8	9 (0)	120 (0)	95 (2)
9	12 (1)	160 (1)	85 (1)
10	12 (1)	80 (−1)	65 (−1)
11	9 (0)	120 (0)	75 (0)
12	6 (−1)	80 (−1)	85 (1)
13	9 (0)	120 (0)	75 (0)
14	6 (−1)	160 (1)	65 (−1)
15	6 (−1)	160 (1)	85 (1)
16	9 (0)	120 (0)	75 (0)
17	6 (−1)	80 (−1)	65 (−1)
18	12 (1)	80 (−1)	85 (1)
19	9 (0)	120 (0)	75 (0)
20	12 (1)	160 (1)	65 (−1)

**Table 2:** Phenolic composition and bioactivity of grape pomace infusions as affected by brewing variables.

Runs	Phenolic Composition				Bioactivity				
	TPC	THC	TFC	TTC	DPPH	LL	BSA	GLU	AMY
1	554 ± 28	68.5 ± 5.3	53.9 ± 5.9	4.26 ± 0.31	1465 ± 16	22.0 ± 1.3	0.367 ± 0.312	571 ± 24	1211 ± 6
2	785 ± 15	52.5 ± 4.5	42.9 ± 5.8	nd	1274 ± 87	29.1 ± 2.0	0.373 ± 0.046	1045 ± 12	85 ± 2
3	789 ± 44	78.3 ± 8.0	52.0 ± 5.5	nd	1575 ± 42	34.0 ± 1.7	0.287 ± 0.032	1115 ± 21	96 ± 7
4	2201 ± 74	112.9 ± 3.5	77.6 ± 5.6	15.40 ± 0.91	4672 ± 326	95.5 ± 0.9	0.919 ± 0.022	1720 ± 70	389 ± 35
5	1936 ± 62	48.1 ± 4.5	72.7 ± 7.2	11.36 ± 0.88	2981 ± 70	52.9 ± 4.6	0.817 ± 0.109	1177 ± 42	233 ± 23
6	1025 ± 39	40.7 ± 4.6	58.5 ± 5.2	nd	2145 ± 98	42.8 ± 3.8	0.515 ± 0.057	1179 ± 20	171 ± 8
7	1093 ± 53	44.5 ± 6.1	59.4 ± 2.1	nd	2033 ± 172	43.9 ± 3.1	0.606 ± 0.018	1161 ± 30	171 ± 5
8	1896 ± 143	54.1 ± 3.4	74.9 ± 7.4	19.30 ± 2.01	3662 ± 266	80.3 ± 5.7	1.679 ± 0,069	1276 ± 55	321 ± 22
9	2901 ± 224	116.4 ± 11.4	84.2 ± 6.3	24.09 ± 0.65	3930 ± 199	76.2 ± 1.1	2.336 ± 0.144	1531 ± 53	302 ± 28
10	985 ± 60	30.5 ± 3.4	64.7 ± 5.1	nd	1849 ± 142	25.7 ± 1.8	1.405 ± 0.121	863 ± 22	125 ± 8
11	953 ± 44	44.5 ± 2.8	62.3 ± 4.4	nd	1988 ± 113	45.0 ± 3.0	0.568 ± 0.040	1207 ± 16	181 ± 2
12	1589 ± 68	51.7 ± 4.3	52.0 ± 7.4	4.49 ± 0.38	1078 ± 185	30.7 ± 1.9	0.578 ± 0.060	913 ± 69	80 ± 1
13	1141 ± 71	45.3 ± 3.9	63.2 ± 5.7	nd	2082 ± 144	42.5 ± 3.6	0.530 ± 0.037	1251 ± 91	181 ± 14
14	1720 ± 26	35.0 ± 2.7	53.7 ± 3.1	15.05 ± 0.36	1252 ± 89	58.0 ± 1.7	0.262 ± 0.023	1390 ± 28	120 ± 10
15	1369 ± 30	46.5 ± 3.5	79.9 ± 2.2	17.23 ± 1.36	3221 ± 91	48.5 ± 1.2	1.268 ± 0.142	1490 ± 16	300 ± 16
16	1052 ± 47	46.9 ± 6.5	60.1 ± 3.1	nd	2116 ± 113	38.3 ± 1.2	0.696 ± 0.074	1202 ± 39	166 ± 5
17	698 ± 24	108.7 ± 2.5	44.6 ± 2.0	2.80 ± 0.14	1126 ± 73	24.6 ± 0.8	0.153 ± 0.048	838 ± 35	129 ± 7
18	2101 ± 54	50.5 ± 0.5	75.1 ± 4.4	9.36 ± 0.55	1468 ± 158	87.7 ± 6.7	0.884 ± 0.069	913 ± 27	201 ± 16
19	1045 ± 44	39.8 ± 2.9	66.3 ± 2.8	nd	2007 ± 121	40.6 ± 0.9	0.514 ± 0.021	1221 ± 51	176 ± 11
20	1845 ± 69	64.5 ± 5.1	64.2 ± 2.1	13.30 ± 0.47	1538 ± 47	62.9 ± 3.4	1.304 ± 0.147	1504 ± 19	232 ± 20

TPC: Total phenolic content as mg gallic acid equivalents L^−1^, THC: Total hydroxycinnamic acid content as mg caffeic acid equivalents L^−1^, TFC: Total flavonoid content as mg rutin equivalents L^−1^, TTC: Total tannin content as mg tannin equivalents L^−1^, DPPH: 1,1-diphenyl- 2-picrylhydrazyl assay as μmol Trolox equivalents L^−1^ infusion, LLA: lecithin liposome assay as μmol rutin equivalents L^−1^, infusion, BSA: bovine serum albumin glycation assay as μmol rutin equivalents L^−1^, GLU: α-glucosidase inhibition assay as μmol rutin equivalents L^−1^, AMY: α-amylase inhibition assay as μmol rutin equivalents L^−1^; nd: not detected.

**Table 3:** Regression coefficient (*β*), coefficient of determination (*R*^2^) and F-test value of the Central Composite Design model for phenolic composition and bioactivity of grape pomace infusions. *β*_1_ = regression coefficient of infusion time, *β*_2_ = regression coefficient of water to solid ratio, and *β*_3_ = regression coefficient of infusion temperature. *p*-values lower than 0.05 are statistically significant and indicated with the symbol *.

Runs	Phenolic Composition				Bioactivity				
	TPC	THC	TFC	TTC	DPPH	LLA	BSA	GLU	AMY
*β*_0_	1119.0	43.66	62.11 *	0.82 *	1953 *	42.53 *	0.631 *	1206.3 *	172.0 *
*β*_1_	296.2 *	−2.52	6.21 *	1.87	426 *	8.03 *	0.295 *	19.06 *	31.56 *
*β*_2_	359.8 *	6.87	5.81 *	4.71 *	646 *	14.00 *	0.203 *	292.84 *	59.69 *
*β*_3_	308.4 *	1.85 *	8.00 *	3.91 *	475 *	10.90 *	0.285 *	44.71 *	46.81 *
*β*_11_	111.1	492 *	0.42	1.83	12,0	0.50	0.025	−12.81	−3.65
*β*_22_	114.8	11.80 *	1.27 *	2.87 *	172,1	4.33 *	0.048	−13.00 *	18.98 *
*β*_33_	105.6 *	2.45	−0.44	2.83 *	59.6 *	3.32	0.143 *	−9.16 *	5.98
*β*_12_	107.0	22.36 *	−3.55 *	0.38	98.0	−3.19	0.069	16.20	0.40
*β*_13_	204.0	14.69 *	−0.40	2.04	124	9.83 *	−0.115	−12.1	1.90
*β*_23_	−163.0 *	12.55 *	3.55 *	0.24	336 *	−8.04 *	0.267 *	0.30	27.90 *
*R* ^2^	0.88	0.93	0.96	0.86	0.86	0.92	0.87	0.99	0.92
*F value* (*model*)	8.26 *	13.80 *	27.04 *	6.59 *	7.00 *	12.40 *	4.53 *	159.34 *	12.44 *
*F value* (*lack of fit*)	40.37	9.98	23.08	1.40	60.95	24.56	44.59	0.99	59.99

TPC: Total phenolic content, THC: Total hydroxycinnamic acid content, TFC: Total flavonoid content, TTC: Total tannin content, DPPH: 1,1-diphenyl-2-picrylhydrazyl assay, LLA: lecithin liposome assay, infusion, BSA: bovine serum albumin glycation assay, GLU: α-glucosidase inhibition assay, AMY: α-amylase inhibition assay.

**Table 4:** Experimental and predicted values for phenolic composition and bioactivity in optimized grape pomace infusion.

Dependent Variables	Composition and Bioactivity		Bioactivity	
	Predicted Value	Experimental Value	Predicted Value	Experimental Value
TPC	2935	2789 ± 189		
THC	187.5	194.0 ± 11.3		
TFC	106.8	102.6 ± 8.8		
TTC	43.65	40.9 ± 3.9		
DPPH	7803	7658 ± 455	8005	7852 ± 389
LL	96.4	100.1 ± 9.3	114.2	108.6 ± 10.1
BSA	3.49	3.34 ± 0.21	3.68	3.60 ± 0.25
GLU	605	621 ± 34	629	622 ± 24
AMY	1800	1844 ±56	1808	1788 ± 60

TPC: Total phenolic content as mg gallic acid equivalents L^−1^, THC: Total hydroxycinnamic acid content as mg caffeic acid equivalents L^−1^, TFC: Total flavonoid content as mg rutin equivalents L^−1^, TTC: Total tannin content as mg tannin equivalents L^−1^, DPPH: 1,1-diphenyl- 2-picrylhydrazyl assay as μmol Trolox equivalents L^−1^ infusion, LLA: lecithin liposome assay as μmol rutin equivalents L^−1^, infusion, BSA: bovine serum albumin glycation assay as μmol rutin equivalents L^−1^, GLU: α-glucosidase inhibition assay as μmol rutin equivalents L^−1^, AMY: α-amylase inhibition assay as μmol rutin equivalents L^−1.^

**Table 5:** Minimum Inhibitory Concentration (MIC) values of grape pomace infusion, gallic acid, catechin and quercetin against foodborne pathogens.

Foodborne Pathogens	Infusion	Gallic Acid	Catechin	Quercetin
	MIC (mg mL^−1^)			
*L. monocytogenes* ATCC 23074 (serotype 4b)	0.5	2.0	2.0	0.5
*L. monocytogenes* EGD (serotype 1/2a)	>2	0.5	0.125	1.0
*L. monocytogenes* Scott A (serotype 4b)	0.5	1.0	2.0	0.5
*L. monocytogenes* NCTC 7973 (serotype 1/2a)	0.5	2.0	2.0	0.5
*L. monocytogenes* NCTC 4885 (serotype 4b)	1.0	2.0	2.0	0.5
*L. monocytogenes* NCTC 4994 (serotype 4b)	1.0	2.0	0.5	1.0
*L. monocytogenes* NCTC 1792 (serotype 4b)	0.25	2.0	1.0	0.5
*Salmonella enterica* subsp. *enterica* serovar Enteritidis NCTC 5188	1.0	2.0	2.0	1.0
*Staphylococcus aureus* ATCC 6538	>2	2.0	0.125	1.0
*E. coli* ATCC 11775	2.0	2.0	>2	0.5

## Data Availability

Data is contained within the article.
